# Ready, set, co(produce): a co-operative inquiry into co-producing research to explore adolescent health and wellbeing in the Born in Bradford Age of Wonder project

**DOI:** 10.1186/s40900-024-00578-y

**Published:** 2024-04-30

**Authors:** David Ryan, Hannah Nutting, Chloe Parekh, Suzie Crookes, Lauren Southgate, Kenzie Caines, Phoebe Dear, Abel John, Muhammed Adnan Rehman, Dawn Davidson, Usayd Abid, Lewis Davidson, Katy A. Shire, Rosemary R. C. McEachan

**Affiliations:** 1 Bradford Institute for Health Research, Bradford Teaching Hospitals NHS Foundation Trust, Bradford Royal Infirmary, Duckworth Lane, Bradford, BD9 6RJ UK; 2 AllStar, Park View Court, St Pauls Road, Shipley, BD18 3DS UK; 3 Bradford District Care NHS Foundation Trust, New Mill, Victoria Road, Saltaire, Bradford, BD18 3LD UK

**Keywords:** Co-production, Young people, Co-operative inquiry, Experiential expertise, Reflective accounts

## Abstract

**Background:**

Co-production of research with communities and stakeholders is recognised as best practice, but despite this, transparent reporting and reflective accounts on co-producing research is lacking. Born in Bradford Age of Wonder (AoW) is a large longitudinal health research project, following the health trajectories of up to 30,000 young people across the Bradford district; moreover, AoW has been entirely co-produced with teachers, parents, and young people. This paper describes the co-production of the Born in Bradford Age of Wonder (AoW) project and shares general reflections on co-production from peer researchers involved in co-producing AoW.

**Methods:**

A co-operative inquiry (CI) approach was used to gather written reflections on co-production from ten peer researchers (one teacher, one parent, eight young people) involved in co-producing the AoW project. Written reflections were collected and rough “themes” were identified using thematic analysis.

**Results:**

Four key ‘themes’ were identified: (1) promoting young people’s voice and views (2) identifying impacts of co-production, (3) fostering a collaborative ethos, and (4) suggested improvements to the co-production work in AoW. Peer researchers’ reflections highlighted how co-production can positively impact research projects such as AoW, whilst also holding broader benefits including giving young people a voice, facilitating their personal development, and fostering a collaborative ethos both within AoW and with partner organisations. Suggested improvements to AoW co-production included supporting greater numbers of young people and researchers to engage in co-production, organising more regular sessions, and establishing clearer communication channels.

**Conclusions:**

Peer researchers’ reflections highlight positive impacts of engaging in co-production, both for research projects (including AoW) and for peer researchers’ personal and professional development. That said, continued efforts are needed in AoW to meet young people’s needs and interests, maintain trusting relationships, and foster sustained growth of co-production efforts within and beyond the AoW project. Evaluation of AoW co-production, along with wider partnership building are key to these efforts.

## Abstract (plain language summary)

Born in Bradford (BiB) is a large health research programme, working to improve the health and wellbeing of people in Bradford and beyond. BiB Age of Wonder (AoW) is the next stage of the BiB programme and is collecting data on up to 30,000 teenagers across the Bradford district. A key part of BiB research (and AoW) involves working with community members as equal partners, through a process called co-production. Co-production is often seen as the best way to do health research; however, not all researchers agree on important questions such as what co-production really is, or why or how it is done. To answer these questions, we need to better understand the perspectives and experiences of those involved in co-production. This study gathered written reflections on co-production from young people, teachers and parents (described as peer researchers) involved in co-producing AoW. The study looked to capture peer researchers’ experiences of doing co-production in general, what possible impact it has, and how co-production in AoW can be improved moving forward.

Findings indicated that taking part in co-production can help peer researchers directly impact projects (including AoW), gain useful skills, and encourage collaboration within and beyond AoW. Suggested improvements to AoW co-production included having more regular sessions and having clearer communication with peer researchers. Whilst these findings indicate that AoW co-production is generally working well, an important next step is to evaluate the AoW co-production work and highlight key successes and challenges.

## Background

Described as a ‘kind revolution’ [1], recent years have seen a growing endorsement of co-production in research [2], healthcare [3], parliamentary/local authority settings [4] and grassroots level organisations [5], while co-production is often described as ‘best practice’. That said, there remains ongoing discourse regarding (1) what constitutes co-production, (2) why co-production is worthwhile, and (3) how co-production can be done effectively, particularly in research. Transparent reporting and reflective accounts (particularly from peer researchers/non-academics) are needed to help answer these questions.

Born in Bradford Age of Wonder (AoW) is a longitudinal mixed-methods research project, following the health trajectories of up to 30,000 young people across the Bradford district. Co-production with teachers, parents, and young people underlies all aspects of the AoW project. Recognising the need for more transparent reporting, the present study brings together academic and peer researchers involved in co-producing the AoW project and uses a co-operative inquiry approach to reflect and transparently report on peer researchers’ experiences of co-production.

### What is co-production in research?

Definitions of co-production are often vague and numerous [6, 7]. The National Institute for Health and Care Research (NIHR) define co-production as an approach in which ‘researchers, practitioners and members of the public work together, sharing power and responsibility from the start to the end of the project, including the generation of knowledge’ [7–9]. Others have described co-production as part of a research involvement continuum, from consultation through to co-production [8]. However, it isn’t always clear what differentiates different types of involvement from each other [8–11]. Williams et al. [11], have termed this the ‘cobiquity’ phenomenon where various co-words (e.g. co-production, co-design) are used interchangeably; this may breed confusion amongst researchers on what constitutes co-production and lead some to conflate meanings and associated practices [12]. A number of reviews have looked to address this confusion, exploring the use of co-production and/or co-design approaches in school-based health interventions [13], in health and social care [14], and in knowledge mobilisation of health conditions [15]. A common finding across the three reviews was the utility of conceptualising co-production as a set of process, functions [13] and/or principles and values [14, 15]. Together, these reviews point towards a conceptualisation of co-production as an enactment of key values and principles, rather than an adherence to a prevailing definition.

### Why do co-production in research?

There are several reasons why researchers engage in co-production. Previous evidence suggests practical benefits such as positively impacting how the research is conducted, and its subsequent impact [16, 17]. For example, involvement of ‘experts by experience’ in research can help produce research that is more relevant to the public and to public needs, subsequently informing better services, interventions, treatments etc [18]. Co-production may also be a generative space, whereby fresh and new ideas can be co-created [10]. Other reasons for co-production include a more democratic and/or moral perspective, encouraging public involvement and co-production because it is the right thing to do, and helps to devolve traditional power dynamics between researchers and community members [11]. In the case of research with young people, co-production can also facilitate shared decision-making between adults and young people on topics relevant to both groups [19].

### How to do co-production in research?

Another important question to answer is how to do co-production. Price et al. [8] outline how co-production may be integrated along various parts of a research project, including setting priorities and developing research questions, building recruitment plans, piloting measures, disseminating findings, and identifying next steps. However, current understanding regarding what works for whom and in what context remains inadequate [20, 21]. This is partly due to a lack of transparent reporting [21–25], highlighting a need for researchers to reflect and transparently report on their experiences carrying out co-production work, including both successes and challenges/failures [26].

In terms of co-producing research with young people, key challenges for facilitators may include (1) managing their time in order to recruit and train co-production group members [27], (2) managing the additional administrative workload associated with group development and maintenance [28], (3) training peer researchers without over-professionalising them [29, 30], (4) developing and maintaining long-term relationships with group members and minimising dropout [20, 31], (5) learning effective facilitator skills and making content accessible and engaging [28], (6) managing differing expectations, viewpoints and/or timelines across research and co-production groups [28], (7) having flexible working hours (i.e. evening/weekend sessions), (8) acting as a gatekeeper for co-production groups, (9) safeguarding young people and protecting them from harm [30, 32].

For project leads, key challenges of embedding co-production effectively may include (1) allocating sufficient time and budget to enable effective co-production [27], and (2) ensuring smooth succession plans if facilitators change or leave roles [28]. For peer researchers, particularly young people, some barriers to active involvement may include researchers’ overuse of academic jargon, timing conflicts with school and/or after-school clubs, and a relative lack of control in their own participation [26]. For example, young people typically need parental consent to take part in co-producing research and young people may need support from parents to attend in-person sessions. Moreover, communication channels often go from researchers to parents in the first instance, as such young people can be reliant on parents to keep them informed of upcoming meetings and project developments. However, despite the many challenges inherent to this type of work, as Kelly et al. state, these challenges are *“far outweighed by the benefits of the peer research approach.”* [27].

### Context: co-producing BiB Age of Wonder

Born in Bradford is a multi-ethnic birth cohort in Bradford, UK which recruited 12,453 women with 13,776 pregnancies and 3448 of their partners between 2007–2011 [33]. It aims to understand why some families stay healthy and why others fall ill. Since its inception, BiB has looked to inspire change by coupling large scale data collection with active community engagement and co-production. BiB Age of Wonder (AoW) signals the next phase of the programme and is collecting data on up to 30,000 young people (including the original BiB cohort) across the Bradford district. AoW looks to work with schools, families, and young people to understand young people’s health and wellbeing trajectories through the sometimes tumultuous periods of adolescence and early adulthood. AoW is also entirely co-produced, following in a tradition of people-powered, co-produced research at Born in Bradford [20, 34–36].

From the outset, key aims of AoW co-production included setting priorities, developing methods and study materials, and later supporting dissemination and impact [37, 38]. Through the first three years of the project (and at the time of writing), AoW co-production work has included a total of 96 sessions with 12 separate groups, including groups of young people (secondary school students and school leavers), parents, and teachers. Of those original groups, six key groups have continued throughout all three years of the project and at the time of writing are the key co-production groups. Co-production work has been embedded into all stages of the AoW project and peer researchers have had direct involvement in activities such as setting priorities, revising and refining AoW study documents (e.g. information sheets, consent forms, questionnaire measures) supporting recruitment to co-production groups, promoting AoW through promotional videos and an art exhibition, and presenting AoW findings at collaborator meetings and festivals.

**Fig. 1:**
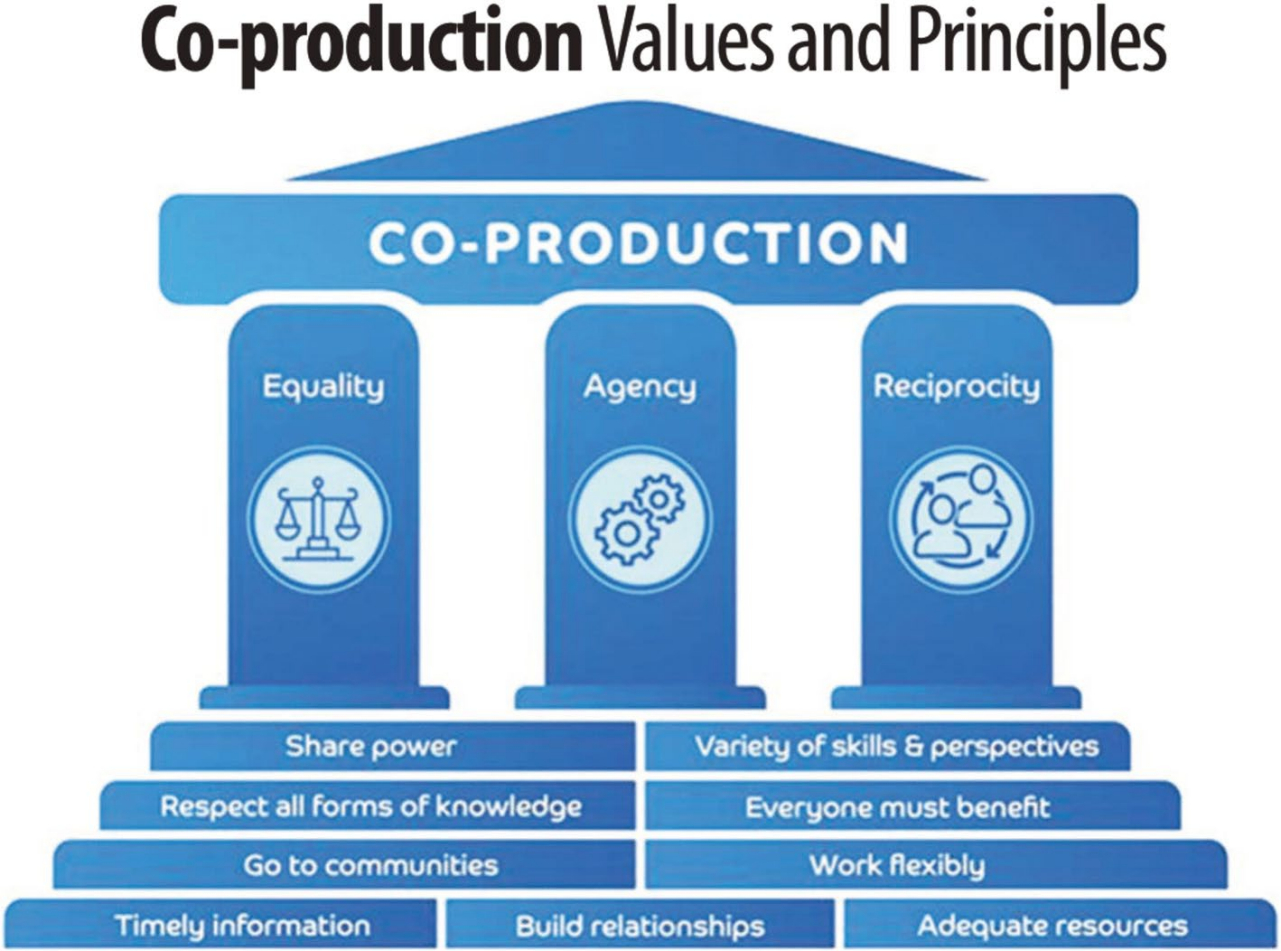
Three core values (equality, agency, reciprocity) and nine key principles as outlined in the ActEarly co-production strategy [20, 39]

The approach to co-production in AoW is guided by the core values and key principles outlined in the ActEarly co-production strategy [20, 39] linked to the Born in Bradford programme. The ActEarly strategy includes nine key principles acting across three core values of equality, agency, and reciprocity (see Fig. 1). Equality and agency are promoted in AoW through measures such as coming to group decisions on session style and format (e.g. length of sessions, having sessions online or offline, use of powerpoints or not, use of pre/post session materials, inclusion of breaks, choice of refreshments), regularly requesting group feedback on sessions, and where possible, aligning the focus of sessions to different groups’ core interests (e.g. working with healthy minds apprentices on mental health component of AoW project). Other measures include agreeing actions as a group at the end of sessions; at the start of the next session, these actions are revisited, and a progress update is given by the facilitator. Reciprocity is promoted in AoW through measures such as providing vouchers for group members’ attendance, providing refreshments for in-person sessions, acknowledging peer researchers in relevant outputs, and providing upskilling opportunities to young people through project-related activities (e.g. presenting AoW findings at the 2023 Born in Bradford festival), work experience and short placements with the AoW team [38].

### Aims

This study aimed to better understand peer researchers’ experience of co-producing research, by gathering reflections from peer researchers involved in co-producing the Born in Bradford Age of Wonder project. In using a co-operative inquiry approach [40], this study also takes on the suggestion by Scholz and Bevan [41] to engage in reflexivity with public participants, in this case peer researchers from AoW co-production groups. The paper also adheres to the GRIPP2 checklist [42] for reporting public involvement in research (see Appendix).

## Methods

### Co-operative inquiry approach

**Fig. 2:**
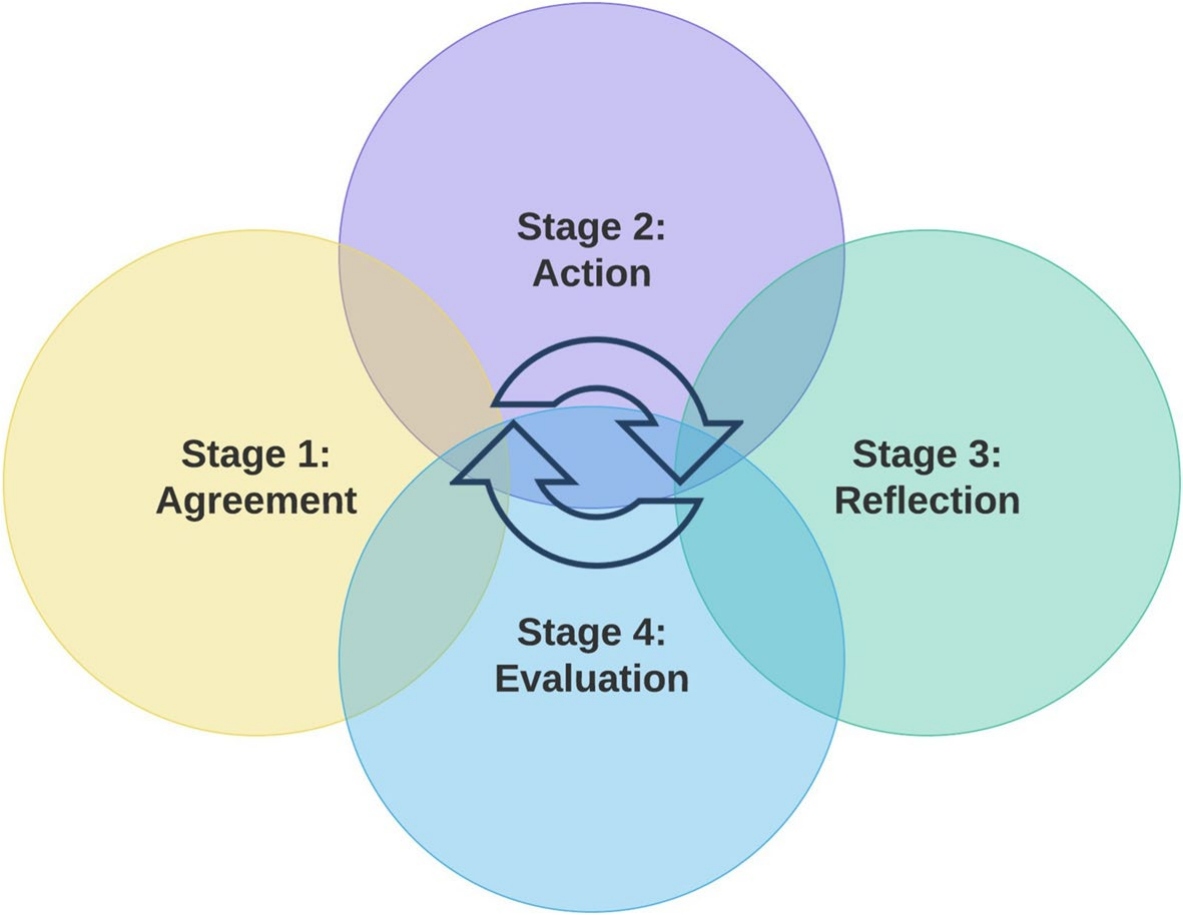
Four stages of the co-operative inquiry process

This paper used a co-operative inquiry (CI) approach [40]. CI approaches have previously been applied in academic [43], healthcare [44–46], and education [47] settings and typically involve a four-stage iterative process (agreement, action, reflection, evaluation) (see Fig. 2). Members of the inquiry may repeat these stages until such time that they are satisfied that the inquiry has reached its natural conclusion. CI can be defined as “an approach to learning and inquiry that combines research and practice for the purpose of transformational change” [48]. The CI approach shares a similar ethos to co-production, in that it emphasises doing research with people, rather than to people. With a CI approach “the split between "researcher" and "subjects" is done away with, and all those involved act together as "co-researchers"” [40]. These steps help promote equality within the team and help reduce potential power imbalances. Moreover, all parties are involved in the activity being researched (in this case AoW co-production) and collectively agree on what questions are asked and what conclusions are drawn.

### Step 1. Agreement

All members of AoW co-production groups (*N* =  ~ 60) were invited to share their thoughts and reflections in the co-operative inquiry, to provide an equality of opportunity and gather a variety of perspectives. Invitations were sent via email, and during regular group sessions held either online or in person in early 2023. During group discussions in Spring 2023, interested members co-designed the initial concept for this study, including the focus of the inquiry, desired level of peer involvement, mode of dissemination, and appropriate timeframes for completion. During subsequent sessions, the scope and ambitions of the co-operative inquiry were sketched out further and group members were given the opportunity to ask further questions. A set of six questions was co-designed, to assist peer researchers in formulating their thoughts and reflecting on their experiences. These questions were drafted by author CP, and during subsequent discussions, authors DR, HN, and CP refined these questions, consulting co-production groups in the process.

1. What does co-production mean to you?
2. What is your experience with co-production?
3. What do you gain from co-production?
4. Why is co-production important?
5. What is important to look at during co-production sessions?
6. How would you improve co-production?

### Stage 2. Action

Once these questions were finalised, they were sent out to all groups via email in May 2023. This email outlined the aim of the study, the ask for peer researchers, and what would happen to their responses. In order to minimise any feelings of coercion or undue pressure (particularly for young people involved in co-production groups), peer researchers were invited to self-select if interested in taking part. A google form was distributed, to host all written reflections, and facilitate shared working. The email reiterated how long the agreed writing window would be (six weeks). Follow-up emails were sent at two and four week points. DR corresponded with peer researchers via email and during regular group sessions, answering any queries people had regarding the aims and scope of the study, and their level of involvement. At the end of the action stage, a group of two academic researchers (HN, DR) and ten peer researchers had been self-selected. Peer researchers were broadly representative of the co-production groups overall, and included eight young people (CP, LS, KC, PD, AJ, MAR, UA, LD), one teacher (DD) and one parent (SC) representing five co-production groups involved in AoW. The peer researcher group also consisted of six females and four males, one Black British researcher, six White British researchers, and three South Asian researchers.

### Stage 3. Reflection

Throughout the reflection stage, dedicated time was ringfenced during monthly co-production sessions so that peer researchers could (1) update on progress, (2) ask questions, and (3) discuss their experiences and reflections. DR also corresponded with peer researchers in-person and via email during this stage, to discuss their experiences of co-production and their experience doing written reflections. DR, HN and CP also met bi-weekly during this time to discuss the written reflections and their experiences of co-production.

### Stage 4. Evaluation

Once the writing window had closed, all responses were collected and authors DR and CP read through each response. NVivo 14 software was used to store and organise all written reflections. In order to better understand peer researchers’ views on co-production, and to find common ‘themes’, CP and DR followed the steps of thematic analysis (familiarisation, generating initial codes, searching for themes, reviewing themes, defining and naming themes, producing the report) as outlined by Braun & Clarke [49]. CP led the analysis, with support from DR. Given the exploratory nature of the inquiry, thematic analysis was largely inductive in approach. Initial results were shared with the co-authorship group to provide space for suggested changes or additions to the original questions. The original questions remained unchanged; however, the authorship group did revise the mode of dissemination, opting to disseminate the findings from the inquiry both as a written piece, but also as a short-form podcast. Moreover, some peer researchers assumed greater responsibility during the evaluation stages (e.g. CP assumed greater coordination and reflection duties), whilst others opted to finish their reflections sooner due to time constraints etc. Initial drafts of the manuscript were sent to all peer researchers for initial review. Two additional members of the team, who initially developed the research ideas (KS, RMc) and the ActEarly co-production strategy (RMc), were invited at this point to review and contribute to the manuscript. Prior to submission, the finalised manuscript was circulated to all co-authors for final comments and sign off.

## Results

**Fig. 3:**
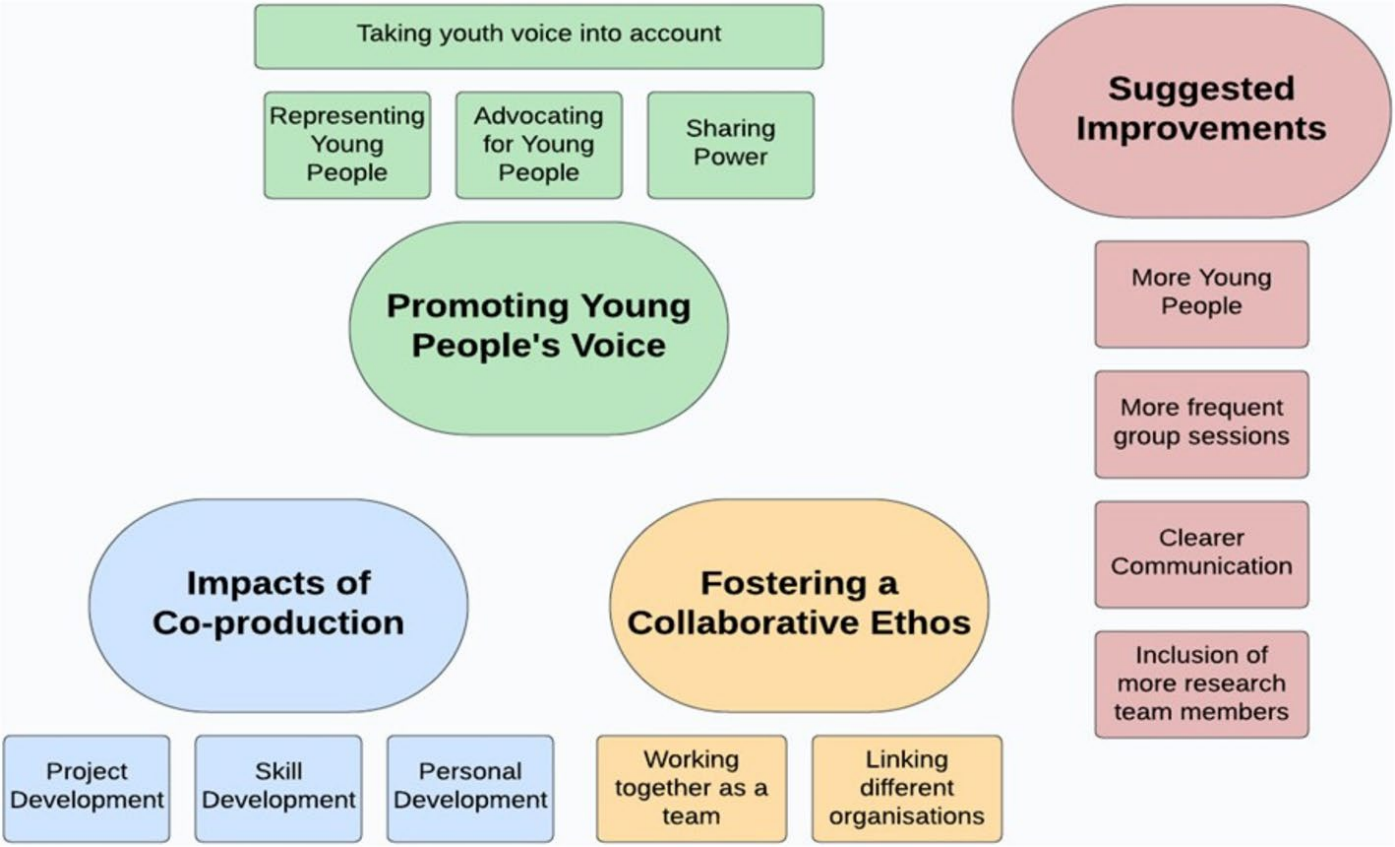
Four key ‘themes’ and associated sub-themes identified from the co-operative inquiry

Four key ‘themes’ were identified from the co-operative inquiry: (1) promoting young people’s voice, (2) impacts of co-production, (3) fostering a collaborative ethos, and (4) suggested improvements. The following section will discuss these themes, with supporting excerpts from the peer researchers (see Fig. 3).

### Promoting young people’s voice

Promoting young people’s voice was highlighted as an important aspect of co-production. Within this theme, youth representation, advocacy, and taking youth voice into account were named as particularly salient. For example, DD mentioned how “*co-production is all about working together, involving others and listening to the views of all, it is about the voice of many rather than that of a few, it is about bringing together the ideas from different viewpoints and producing something that is fit for purpose”* and that co-production facilitates* “an inclusive, varied view from those who matter the most*”. Similarly, MAR spoke about how co-production *“allows the sharing of diverse views and ideas and getting a representative input from different perspectives”,* while LS described how “co-production is important because it allows for those who can’t usually get their voice heard to speak out, change services and share their experiences”.

CP summarised her experience of co-production as *“a really good working and trusting relationship, as we are able to voice our opinions and not feel like anything we say is wrong or will be judged. During co-production sessions I feel listened to and I am able to talk freely, within reason, on related topics in order to make a difference.”* Reflecting on co-producing AoW and the role of young people, CP mentioned how* “it is important to involve young participants in anything that is tailored towards young people, as they understand what works for the majority of young people and they have more of an idea on what wouldn’t work for some young people, ensuring that it is tailored to everyone’s, if not, most people’s needs.”*

When discussing youth voice, a number of peer researchers stressed an additional need: actively taking that youth voice into account. For example, KC mentioned how *“during co-production everyone is seen as an equal and all thoughts and beliefs are valued. During co-production sessions I feel that my voice is listened to and valued.”* This sentiment was echoed by CP: *“Co-production can help us to challenge the idea that individuals with lived experience aren’t able to participate on an equal level with people in professional roles. It’s also an exceptionally effective way for people with lived experience to be able to influence change for young people…understanding young people’s priorities ensures that researchers are able to look at ways of improvement. It allows young people to have a focus on things that they believe need to be changed, and enabling them to voice their opinions, concerns or questions allows them to feel empowered and to feel like anything they may suggest will be taken seriously, as a result of the relationship built within the co-production sessions.”* Whilst peer researchers highlighted the importance of taking youth voice into account, MAR stressed how “*it is important to look at realism, practicalities, and viability. We may have a wish list for co-production but we need to ensure that suggestions are achievable.*”

### Impacts of co-production

In their reflections, peer researchers mentioned some positive impacts of engaging in co-production, both for research projects and for those involved in co-production. LD mentioned how co-production gives projects “a broader view”, while UA stated how co-production *“makes sure that a problem can be tackled from all angles. This ensures maximum efficiency and accuracy when it comes to releasing information to other researchers and the public. It can also prevent potential mistakes in the form of mono-view approaches and allow for alternative viewpoints that may otherwise be missed. If done correctly, it can greatly improve the outcome of the project.”*

Another impact of co-production was the impact on those involved, in terms of personal and professional development. As PD outlined: *“I gained a voice through co-production, it allows me to talk through my experiences and the experiences of other young people and apply it to the work we are doing*.*”* Meanwhile, for UA *“there is a certain satisfaction, knowing that I have contributed (no matter the size). This contribution could be part of a larger piece of research, that leads onto an impact that can positively influence someone's life.”*

LS mentioned how *“you gain valuable communication and people skills from co-production as it’s all about cooperating with clients and colleagues to achieve the goal set for the resource in question”*, AJ mentioned how *“co-production allows people to communicate with others and work as a team which provides valuable experience for future job prospects”*, while MAR spoke about how he *“was able to gain confidence in sharing my ideas, listening to others and working jointly in collaboration towards an end product.”* Finally, CP described how co-production enabled young people to select how they wanted to be involved: *“Having opportunities such as these available for young people who are involved in aspects of the planning creates an incentive for young people to fully engage in co-production and allows them to enjoy and partake in activities and fulfil roles they want to achieve.”*

### Fostering a collaborative ethos

Another theme identified in peer researchers’ reflections was the collaborative ethos inherent in co-production work. For peer researchers, this collaborative ethos was present within groups, but also extended to collaborations between organisations. DD mentioned how *“co-production is all about working together, involving others and listening to the views of all….it makes discussion valuable.”* AJ described co-production as *“working together as a team in order to produce a final product which represents the hard work of the team.”*

Some peer researchers dove a little deeper, highlighting what helps to facilitate active collaboration, as CP describes: *“Activities within co-production are very useful, as not only is it engaging young people further, but some young people are more likely to get more out of it and work better in a casual environment. For example, working in small groups and writing down ideas is useful, as young people may be more likely to communicate their ideas better that way, and it gives everyone a chance to write their own ideas. As well as this they are able to learn from other young people too, as they’re communicating their thoughts and ideas.”*

Some peer researchers also considered co-production as part of a broader collaborative effort; for example, CP described how co-production involved communicating with different organisations, not just co-production groups: *“co-production to me is about joining other organisations in order to make a difference and to make changes within services”.* PD shared similar sentiments, seeing this collaboration as cause for optimism:* “During my last 2 years working in the mental health sector, I’ve had many experiences with co-production. A lot of the sessions included working on research, mental health and service reviews where I advocated youth voice. I have found it inspiring and impactful, watching organisations communicate and work together brings hope to the sector.”*

### Suggested improvements

The last question posed to peer researchers explored how co-production in AoW could be improved. The written reflections provided some valuable insights, for example, more sessions were suggested by AJ, who described how he *“would have more frequent meetings in order to track the progress of the work.”* Similarly, LS stated: *“To improve co-production, I would simply say to do more of it with a diverse range of people young and old to better services and make changes.”* Involvement of more peer and academic researchers was also suggested; UA mentioned increasing co-production activities, saying *“I think just ensuring that we can get as many people as possible to contribute. This can be done by trying to get as many people as possible to participate by coming to sessions.”*

CP mentioned that *“a way to improve co-production is by including more people from BiB/AoW to be involved in the co-production, this would help to be able to build better relationships with the people we are working with andl help them to also get first-hand advice/opinions from us”*, DD suggested *“having all subgroups come together at least once and having focused questions set for ideas”*, while PD suggested that *“the only thing that would improve co-production is if more people participated in it and we can assess what work has already been created so we can share resources.”* MAR mentioned that *“whilst I understand that face to face sessions were not possible, I would say that ensuring that sessions are planned in advance, face to face would be advantageous and have time to share ideas and learn from others.”* Finally, clearer communication was highlighted as a potential improvement; SC mentioned improving communication to groups to better support people’s involvement, particularly during busy periods in AoW and associated projects: *“With Age of Wonder and Celebrate and the Bib Fest all going on there are quite a lot of things happening and it would be helpful to understand all of the plans and think about what I can help with most.”*

## Discussion

This paper used a co-operative inquiry approach to share reflections on co-production from peer researchers involved in the first three years of AoW co-production; the results highlighted how co-production can positively impact (1) research projects such as AoW, (2) peer researchers, and (3) different organisations. The results also provide some useful contributions to ongoing discourse on what constitutes co-production, along with why and how research should be co-produced.

In their reflections on what constitutes co-production, peer researchers mentioned working collaboratively as a team, including varied and representative voices, building trusting relationships, and finding and using their voice to influence change. Interestingly, many of the peer researchers’ reflections on what co-production is can be linked back to the core values and key principles outlined in the ActEarly strategy [20, 39] (including sharing power, having a wide range of perspectives, working flexibly, building long-term relationships), indicating that these values and principles have been embedded in the AoW project. These sentiments also link closely to the NIHR principles of co-production, such as sharing power, including all perspectives and skills, and building and maintaining relationships [50], providing additional support to the conceptualisation of co-production as an enactment of key principles and values.

Peer researchers’ reflections also provided some reasons why research should be co-produced. For example, peer researchers mentioned how co-production can make research that is “fit for purpose”, whilst the inclusion of young people as co-producers of AoW helps to ensure the project is “tailored to everyone’s, if not, most people’s needs.” An interesting real-life example of this is development of the AoW survey measures, during which often-used measures were replaced as they were widely rejected by co-production groups [2]. Peer researchers also reported experiencing personal and professional skill development through their engagement in co-production (e.g. gaining confidence, gaining communication skills, collaboration skills). Similar findings have been reported elsewhere [26, 28, 30, 31] and highlight how co-production can positively impact projects, but also facilitate technical and soft skill development for peer researchers.

Looking more broadly, peer researchers described a collaborative ethos within and beyond AoW co-production and highlighted benefits of collaboration in research and other settings (e.g. healthcare service improvement). Previous research asserts that researchers, service providers, local authorities and others can benefit from engaging with young people when looking to impact young people’s health and wellbeing [16–18]. Moreover PD’s reflection on how “watching organisations communicate and work together brings hope to the sector” highlights how organisations are often indirectly linked through mutual connections with co-production groups and with the same young people. It also signals an opportunity to facilitate cross-sectoral working through co-production; however further work is needed to do this more effectively and best support young people and community members to inform change.

Results from this study also contribute to questions of how co-production should be done. For example, peer researchers mentioned things such as working in groups, having a casual environment, and providing opportunities across the project lifecycle for peer researchers so they can have greater agency over their level of involvement. Peer researchers also discussed the importance of supporting young people “*to enjoy and partake in activities and fulfil roles they want to achieve”.* This aligns with the ethos of AoW co-production and the present co-operative inquiry. A real-life example of this was the 2023 Born in Bradford scientific festival; young people from co-production were given an opportunities document before the event, outlining possible ways they could be involved (e.g. presenting findings, chairing panels, designing promotional materials), and how they as young people could benefit from the experience (e.g. CV building, professional development). This enabled interested young people to self-select for opportunities that aligned best with their interests and helped provide them with positive experiences of autonomous working in a research context.

Crucially, peer researchers also suggested ways in which AoW co-production could be improved. For example, peer researchers spoke about wanting more frequent sessions, having more people at the sessions, and bringing the different co-production groups together to work as a larger collective, to inform and observe the iterative development of the project. Another suggested improvement involved communicating the different components of the project more effectively, so that peer researchers could make better informed decisions on how and where they most wanted to be involved. Previous research has called for more transparent reporting of unsuccessful cases and practical learnings from co-production [21, 24, 25]; thus, the suggested improvements reported in this study provide practical improvements for the AoW co-production work, but also contribute to the existing co-production knowledge base. These suggestions also illustrate some of the challenges of doing co-production (e.g. administrative workload associated with co-production, need for clear communication channels between academic and peer research groups) and echo previous claims that co-producing with young people is complex [28, 30]. The reporting of these suggestions, and use of co-operative inquiry in the present study, may hopefully encourage others to transparently report on their experience of co-producing research, discuss key challenges, and avoid a co-production ‘file drawer problem’ [51] whereby only successes are reported.

Whilst indications from this co-operative inquiry were largely positive, more formal evaluation of the AoW co-production work is needed. This joins a number of calls in health research for rigorous evaluations of co-production [21, 52–54], including more quantitative measures [52]. Some researchers have criticised existing evaluation tools for being too prescriptive, recommending that co-production evaluations be more context-specific and garner reflections from all parties involved [31]. Moreover, a lack of young people participating as co-evaluators represents a notable gap in co-production evaluations to date [31]. In light of these recommendations, and the current study findings, it is important that evaluating AoW co-production is a collaborative effort between researchers, young people, and community members. Findings from this study also suggest that the evaluation of AoW co-production may benefit from the inclusion of broader outcomes of co-production, such as skill development and increased confidence. This aligns with previous research recommending that considerations of co-production capture outcomes such as skill development, empowerment of service-users, and acceptance of co-production among policymakers [21, 55].

### Strengths & limitations

Key strengths of this study include the use of a co-operative inquiry approach, and the inclusion of reflective accounts from peer researchers, providing a transparent method of reporting. Another strength of this study is the inclusion of perspectives from young people, along with parents, teachers and academic researchers. In research contexts, young people are sometimes viewed as ‘hard-to-reach’ or challenging to work with [56, 57]; subsequently, reporting of children and young people’s involvement and/or level of involvement is often sparse [26]. Similarly, young people may not be given formal credit as authors of co-produced outputs, a practice which can erode trust and community interest [36]. Thus, the inclusion of young people as peer researchers and co-authors is a particular strength of this work.

This study also has some limitations, for example, peer researchers were self-selected, as such the reflections provided may be from those who are most engaged in the project and who may reflect most positively on co-production. Social desirability [58] and observer biases [59] are also distinct possibilities, as many of the academic and peer researchers knew each other and had pre-existing relationships through the co-production work. Moreover, whilst co-operative inquiries endeavour to reduce or remove potential power imbalances, peer researchers may still have felt that they had less power than the academic researchers. Finally, whilst the co-operative inquiry approach helped give peer researchers greater ownership of the study, the questions and written reflections developed during the inquiry often referred to co-production in a general sense; thus, it was not always clear in the present study which reflections were directly attributable to AoW and which referred to co-production more generally. More AoW specific inferences will be gleaned from evaluating AoW co-production.

## Conclusion

Co-operative inquiry approaches provide a useful method for gathering reflections from peer researchers and transparently reporting on co-production. Indications from the present co-operative inquiry are that engaging in co-production can benefit research projects (including AoW), peer researchers, and different organisations. That said, it is important that AoW co-production efforts continue to grow and improve, in order to maintain peer researchers’ trust and engagement in the project, facilitate effective co-production, and co-produce research that benefits the health and well-being of young people across Bradford and beyond. Important next steps include evaluating AoW co-production, and continued partnership building.

## Data Availability

The datasets used and/or analysed during the current study are available from the corresponding author on reasonable request.

## Appendix

**Table:** Populated GRIPP2 checklist [42] for reporting public involvement in research

Section and topic	Item	Reported on page No
1: Aim	Report the aim of PPI^a^ in the study	10
2: Methods	Provide a clear description of the methods used for PPI in the study	11–14
3: Study results	Outcomes—Report the results of PPI in the study, including both positive and negative outcomes	15–20
4: Discussion and conclusions	Outcomes—Comment on the extent to which PPI influenced the study overall. Describe positive and negative effects	20–23
5: Reflections/critical perspective	Comment critically on the study, reflecting on the things that went well and those that did not, so others can learn from this experience	24

^a^*PPI* Patient and Public Involvement

## Abbreviations

BiB: Born in Bradford
AoW: Age of Wonder
NIHR: National Institute for Health and Care Research
CI: Co-operative Inquiry
DR: David Ryan
HN: Hannah Nutting
CP: Chloe Parekh
LS: Lauren Southgate
KC: Kenzie Caines
PD: Phoebe Dear
AJ: Abel John
MAR: Muhammad Adnan Rehman
DD: Dawn Davidson
SC: Suzie Crookes
UA: Usayd Abid
LD: Lewis Davidson
KS: Katy A Shire
RMc: Rosemary RC McEachan
